# Salicylic Acid-Mediated Silver Nanoparticle Green Synthesis: Characterization, Enhanced Antimicrobial, and Antibiofilm Efficacy

**DOI:** 10.3390/pharmaceutics17040532

**Published:** 2025-04-18

**Authors:** Jingqing Zhang, Yuxu Chen, Yuanyu Xu, Zhimin Zhao, Xinjun Xu

**Affiliations:** School of Pharmaceutical Sciences, Sun Yat-sen University, Guangzhou 510006, China

**Keywords:** green synthesis, silver nanoparticles, salicylic acid, antibacterial activity, antibiofilm activity

## Abstract

**Objectives:** Silver nanoparticles (AgNPs) were synthesized via an easy and rapid biogenic synthesis approach, utilizing the dual capabilities of salicylic acid as both a reducing and capping agent. **Methods:** The characterization of Salicylic Acid-Mediated Silver Nanoparticle (SA-AgNPs) was conducted using a variety of techniques, including ultraviolet-visible spectroscopy, dynamic light scattering, scanning electron microscopy combined with energy dispersive X-ray spectroscopy, transmission electron microscopy, X-ray diffraction, Fourier transform infrared spectroscopy, as well as thermogravimetric analysis paired with differential scanning calorimetry. **Results:** SA-AgNPs demonstrated significant antibacterial properties against both Gram-positive (methicillin-resistant *Staphylococcus epidermidis*, *Staphylococcus aureus*, *Cutibacterium acnes*, methicillin-resistant *Staphylococcus aureus*) and Gram-negative (*Escherichia coli*), with minimum inhibitory concentrations (MICs) of 8, 9, 8, 4, and 6 μg/mL, respectively. At a concentration of 32 μg/mL, SA-AgNPs exhibited 99.9% killing efficiency against *Escherichia coli* (*E. coli*), *Cutibacterium acnes* (*C. acnes*), and methicillin-resistant *Staphylococcus aureus* (MRSA), within 4, 16, and 12 h, respectively. At the same concentration, SA-AgNPs effectively inhibited 95.61% of MRSA biofilm formation. SA-AgNPs induced the leakage of intracellular macromolecular substances by increasing the membrane permeability, which ultimately caused bacterial apoptosis. **Conclusions:** Overall, this study presents a fast and environmentally friendly approach for synthesizing SA-AgNPs, with potential applications as nano antibiotics antibacterial coatings for implantable medical devices and wound dressings.

## 1. Introduction

Bacterial resistance to antibiotics has emerged as a worldwide public health concern, particularly regarding Methicillin-resistant Staphylococcus aureus (MRSA) [1,2,3,4]. Conventional antibiotics’ efficacy has reduced as resistant bacteria evolve [5]. Therefore, new alternative therapies are required to avoid drug-resistant bacterial infections.

Nanoparticles are defined as particles measuring between 1 and 100 nm [6]. Because of their distinct physical, chemical, and biological characteristics, nanoparticles have become increasingly popular in various applications, including antibacterial coatings for medicinal materials [7], cancer diagnosis and therapies [8], drug delivery [9] and imaging [10]. In particular, silver nanoparticles (AgNPs) have taken the leading position in these fields [11]. AgNPs also show promise as a potential strategy for overcoming the antimicrobial resistance challenge [12,13]. AgNPs can be manufactured using chemical [14,15], physical [16,17], and green techniques [18,19]. Green synthesis produces stable functionalized AgNPs by reducing and capping them with plant extracts, plant-derived compounds, or microorganisms [20]. Green synthesis is gaining popularity because of its environmental and economic benefits [21].

Notably, recent studies have demonstrated that phenolic compounds can enhance AgNPs’ bactericidal efficacy by synergizing with silver ions: AgNPs synthesized using proanthocyanidins and cinnamic acid exhibit great bactericidal efficacy against both bacteria and fungi [22,23]. Kaempferol-synthesized AgNPs demonstrated enhanced antibacterial efficacy against MRSA relative to commercial AgNPs and kaempferol individually [24]. AgNPs coated with gallic acid have strong antibacterial properties and low cytotoxicity to normal cells [25]. Curcumin-modified AgNPs exhibit enhanced bacterial membrane binding of silver and Ag⁺ release compared to unmodified counterparts, coupled with increased reactive oxygen species (ROS) generation, collectively driving synergistic antibacterial effects [26]. In contrast to chlorogenic acid alone, AgNPs synthesized with chlorogenic acid showed nearly eight times as much antibacterial activity against Pseudomonas aeruginosa [27]. A minimum inhibitory concentration (MIC) of 1.0 μg/mL was demonstrated by quercetin-mediated synthesized AgNPs with an 8 nm diameter against *Streptococcus* sp., *Escherichia coli* and *Candida* sp. Microorganisms [28].

However, no study has explored salicylic acid (SA)—a natural phenolic acid with intrinsic anti-inflammatory and antimicrobial properties [29,30,31]—as both a reducing and functionalizing agent for AgNPs and systematically investigated its potential to combat MRSA biofilms. While the poor aqueous solubility of SA has historically restricted its practical applications, this limitation is circumvented in the synthesis of AgNPs, where SA does not need to be fully dissolved to serve its intended functions. Salicylic acid, as a natural phenolic acid, can convert Ag (I) ions into Ag (0) [31]. Meanwhile, SA’s bioactive properties contribute to the membrane-disrupting and antibacterial activities of AgNPs, creating a synergistic effect [21]. This approach not only resolves the solubility issue but also transforms it into a beneficial characteristic in nanoparticle-based antibacterial strategies.

In this study, we synthesized AgNPs utilizing salicylic acid as both a reducing and capping agent, optimized the synthesis conditions (ultrasonic time, volume ratio and pH) and characterized it using a range of techniques such as UV–Visible spectrophotometer, transmission electron microscopy, scanning electron microscopy, X-ray diffraction and Fourier transform infrared spectroscopy. In terms of biological activity, we evaluated its antibacterial activity, bactericidal activity, and bactericidal efficiency against various kinds of bacterial strains. Furthermore, the antibiofilm activity of SA-AgNPs against MRSA was examined.

## 2. Materials and Methods

### 2.1. Materials

Silver nitrate (AgNO_3_), commercial AgNPs (≥99.5%, 60–120 nm), and ampicillin were from Shanghai Aladdin Biochemical Technology Co., Ltd. (Shanghai, China); Salicylic acid and sodium hydroxide were from Tianjin ZhiYuan Reagent Co., Ltd. (Tianjin, China). Mueller Hinton II broth (Cation-Adjusted) and Mueller-Hinton agar (MHA) were obtained from Solarbio Technology Co., Ltd. (Beijing, China); Brain Heart Infusion (BHI) Broth and Reinforced Clostridium Agar were purchased from HuanKai Microbial (Guangzhou, China). Trypticase soy broth was from Qingdao Hope Bio-Technology Co., Ltd. (Qingdao, China). Luria–Bertani (LB) broth was from Sangon Biotech (Shanghai) Co., Ltd. (Shanghai, China) Crystal violet (CV) was from Macklin Co., Ltd. (Shanghai, China). Phosphate buffered saline (PBS) was from Thermo Fisher Scientific (Waltham, MA, USA).

Gram-negative bacterial strains: *Escherichia coli* (*E. coli*, ATCC25922), Gram-positive bacterial strains: *Staphylococcus aureus* (*S. aureus*, ATCC25923), methicillin-resistant *Staphylococcus aureus* (MRSA, ATCC43300), methicillin-resistant *Staphylococcus epidermidis* (MRSE, ATCC35984) and *Cutibacterium acnes* (*C. acnes*, ATCC6919) were obtained from Guangdong Microbial Strain Preservation Center (Guangzhou, China).

### 2.2. Green Synthesis of SA-AgNPs

A solution of 7.24 μmol/mL SA in ultrapure water (H_2_O) was mixed with 10 mmol/mL AgNO_3_. After adding sodium hydroxide to correct the pH, the liquid was sonicated for 12 min with an ultrasonic power of 360 W and a frequency of 40 kHz. The solution was observed to change from colorless and transparent to dark brown, signaling the creation of AgNPs [32].

### 2.3. Study of the Effect of Different Factors on SA-AgNPs Biosynthesis

#### 2.3.1. Effect of Ultrasonic Time

The experimental conditions were optimized for ultrasonic time, volume ratio, and pH, with the samples’ surface plasmon resonance (SPR) absorbance and particle size serving as reference indexes.

The volume ratio of AgNO_3_:SA was 2:1, and the pH was maintained at 9. To evaluate the impact of ultrasonic time on AgNP synthesis, the samples were exposed to various ultrasonic times (4 min, 8 min, 12 min, 16 min, and 20 min)

#### 2.3.2. Effect of Volume Ratio

The ultrasonic time was 12 min, and the pH was maintained at 9. Different volume ratios of AgNO_3_:SA (1:2, 1:1, 2:1, 3:1, and 4:1) were used to assess the influence of volume ratio on AgNP synthesis.

#### 2.3.3. Effect of pH

It was efficient in lengthening the steady dispersion time of AgNPs by reaching the optimal pH [33] AgNO_3_:SA was fed at a 2:1 volume ratio, and the ultrasonic period was 12 min. The reaction solution’s pH was modified to 6, 7, 8, 9, and 10 to examine the impact of pH on the synthesis of AgNPs.

### 2.4. Characterization of SA-AgNP

A UV-visible spectrophotometer (UV-Vis) (UV-2600, Shimadzu, Kyoto, Japan) was used to measure absorbance from 300 nm to 800 nm, confirming AgNP biosynthesis [34].

The samples were dispersed in water. A Zetasizer Nano ZS instrument (Malvern Instruments, Malvern, UK) was used to measure the polydispersity index (PDI), mean hydrodynamic size, and zeta potential.

To determine the shapes, sizes, and particle morphology of SA-AgNPs, the samples were diluted using ultrapure water and dripped on a carbon-coated copper film until dry at room temperature. The images were taken with transmission electron microscopy (TEM (JEM-2010HR, JEOL, Tokyo, Japan)) at 200 kV accelerating voltage.

The surface morphology and elemental composition of SA-AgNPs were analyzed using SEM (EVO MA10, ZEISS, Oberkochen, Germany) at 5 kV and EDX (Bruker, Berlin, Germany). The SA-AgNPs’ surface morphology was photographed and documented at different magnifications. The EDX result revealed the silver composition percentages in SA-AgNPs.

To analyze the crystalline structure of SA-AgNPs, the powder was flattened into a thin film on a slide, and the X-ray diffraction (XRD) (Empyrean Alpha 1, Malvern Panalytical, Malvern, UK) was operated in the 2θ region (10° to 80°) at a rate of 0.02°/min.

Nitrogen was used as a protective gas, and thermogravimetric analysis paired with differential scanning calorimetry (TG-DSC) (STA449F3, NETZSCH, Selb, Germany) spectra were recorded for SA-AgNPs heated from 33 °C to 900 °C.

The functional groups in SA-AgNPs were identified through Fourier transform infrared spectroscopy (FTIR) (Spectrum two, Perkin Elmer, Springfield, IL, USA), which scans ranging from 4000 to 400 cm^−1^ at a resolution of 0.5 cm^−1^.

### 2.5. Assay of Salicylic Acid

The assay of salicylic acid was conducted based on the approach detailed in prior studies, with certain modifications [35,36]. SA content in SA-AgNPs was evaluated indirectly using reversed-phase high-performance liquid chromatography (RP-HPLC) (LC-20AD, Shimadzu, Kyoto, Japan) on a C18 column (250 mm × 4.6 mm, COSMOSIL). The mobile phase consisted of a buffer solution made from sodium dihydrogen phosphate, with its pH fine-tuned to 2.2 using phosphoric acid, combined with methanol in a 50:50 volume ratio. Prior to application, it was passed through a 0.22 μm membrane filter utilizing a vacuum filtration setup and degassed in a sonication bath. A stock solution of 1 mg/mL SA was made using HPLC-grade methanol. Furthermore, reference substance solutions of 0.01 to 0.15 mg/mL were then generated from the stock solution using methanol as a diluent. The reacted SA-AgNPs solution was centrifuged to obtain a clear supernatant. The supernatant was subsequently passed through a 0.22 μm membrane filter to eliminate any remaining silver nanoparticles. Once filtered, the supernatant, which contained unreacted SA (not interacting with Ag^+^), was separated and acidified with sulfuric acid. Test samples were obtained by diluting the acidified supernatant with methanol. After filtering through a 0.22 μm membrane, the samples were measured at 310 nm. The SA content in the supernatant was measured in triplicate (*n* = 3) using the equation below.

(1) $$\omega =\frac{\rho \times V}{m}\times 100\%$$

where *w*, *ρ*, *V*, and *m* are the mass fraction of SA in the sample (%), SA concentration from the standard curve (mg/mL), test sample volume (mL), and total mass of SA (mg).

### 2.6. Assay of Silver Ions

The silver ions content was determined by inductively coupled plasma-atomic emission spectroscopy (ICP-AES) (Optima8300, PerkinElmer, Springfield, IL, USA) [37,38]. A certain amount of SA-AgNPs powder was dissolved in a 5% *v*/*v* nitric acid solution to prepare silver ion solutions at concentrations between 4 and 8 mg/L. The test samples were then analyzed by ICP-AES, and the silver ion content in SA-AgNPs was calculated using the equation below.

(2) $$\omega =\frac{c\times V}{1000\times m}\times 100\%$$

where *w*, *c*, *V*, and *m* are the mass fraction of silver in the sample (%), silver concentration from the standard curve (mg/L), test sample volume (mL), and sample weight of SA-AgNPs (mg).

### 2.7. Stability of SA-AgNPs

To evaluate the stability of SA-AgNPs, the samples were kept in a refrigerator at 4 °C, shielded from light exposure.

Over specific time intervals—0, 2, 5, 7, 14, 28, 35, 49, and 65 days—the particle size, polydispersity index (PDI), and zeta potential were measured. Additionally, UV-Vis spectroscopy was employed to scan the samples across a wavelength range of 300–800 nm [38].

### 2.8. Antimicrobial Activity of SA-AgNPs

#### 2.8.1. Minimum Inhibitory Concentration and Minimum Bactericidal Concentration (MIC and MBC)

The broth microdilution assay was performed in a 96-well plate to assess the minimum inhibitory concentration (MIC) and minimum bactericidal concentration (MBC), following the Clinical and Laboratory Standards Institute (CLSI) guidelines (M07) [39]. SA-AgNPs, commercial AgNPs, and ampicillin dispersions were all formulated to a specific concentration employing ultrapure water, while SA dispersions were prepared with 20% ethanol. The bacterial suspensions were adjusted to the appropriate optical density at 600 nm (OD_600_) and then spread onto Mueller-Hinton agar (MHA) plates. These plates were incubated at 37 °C for a period of 24 h to verify that the concentration of the bacterial suspensions reached 1 × 10^8^ colony-forming units (CFU)/mL.

The bacterial suspensions (1 × 10^8^ CFU/mL) were diluted one hundred-fold with cation-adjusted Mueller-Hinton broth (CAMHB), and BHI medium was used for *C. acnes*. Finally, each well contained 100 μL of diluted bacterial dispersions and 100 μL of sample dispersions (SA-AgNPs, commercial AgNPs, SA, ampicillin, and 20% ethanol) for a final volume of 200 μL. Sterile CAMHB served as the negative control, ampicillin as the positive control, and 20% ethanol as the solvent control.

The 96-well plates were placed in an incubator at 37 °C for 24 h. (72 h for *C. acnes*). *C. acnes* were incubated under anaerobic conditions using an AnaeroPack (Japan Mitsubishi MGC, Saitama, Japan). The results were visually assessed, with the MIC determined as the lowest dose at which no bacterial growth could be detected with the naked eye. To determine the MBC, 100 μL of the mixture from MIC wells showing no bacterial growth was then transferred and cultured on MH agar plates, incubated at 37 °C for 24 h.

#### 2.8.2. Time-Kill Assay

CLSI guidelines (M26h) [40] were followed for performing Time-Kill assays. Bacteria were inoculated into sterile CAMH broth (BHI medium for *C. acnes*) to achieve a final concentration of 1 × 10^6^ CFU/mL. Diluted bacteria suspensions were added to each tube containing SA-AgNPs in sterile CAMH broth. Time-kill assays were conducted by applying final concentrations of SA-AgNPs at 1/2 × MIC, 1 × MIC, 2 × MIC, and 4 × MIC levels. After incubation at 37 °C, 100 μL of the diluted mixture was cultivated on MH agar plates (Reinforced Clostridium agar plates for *C. acnes*) at each time point. The plates were subsequently incubated at 37 °C for 24 h (72 h for *C. acnes*). The colonies on each plate were counted to assess the bacterial concentration at each time point.

Sterilization was regarded as complete when the bacterial concentration reached one-thousandth of the initial concentration.

### 2.9. Antibiofilm Ability Studies

#### 2.9.1. Inhibition of Biofilm Formation

The biofilm formation of MRSA was assessed in 96-well microplates using microtiter plate assay, following a previously reported method with modifications [41]. Bacterial suspension diluted in Trypticase soy broth (TSB) with 1% (*w*/*v*) glucose and varying sample concentrations were transferred to each well of the 96-well plates. Positive controls consisted of wells containing only TSB and MRSA, while negative controls were wells filled solely with sterile phosphate-buffered saline (PBS, pH 7.4). The plates were then incubated at 37 °C for 48 h. Following incubation, the contents of each well were carefully discarded, and the wells were rinsed three times using 300 μL of PBS. Once the plate was dried, the biofilms on the bottom of the wells were fixed with 150 μL of 95% methanol. To visualize the biofilms, 0.1% crystal violet (CV) was introduced and left to incubate for 15 min at room temperature. Excess CV was removed by washing the wells with 300 μL of sterile distilled water until the wash solution ran clear. Subsequently, 200 μL of 33% glacial acetic acid was added to each well to dissolve the CV bound to the stained biofilm. A 150 μL aliquot from each well was then transferred to a fresh 96-well plate for further tests. The absorbance of each well was measured at 562 nm using a microplate reader to quantify biofilm formation. The cutoff optical density (ODc) was determined as the mean OD of the negative control plus three standard deviations [42]. The percentage of biofilm inhibition was then calculated using the following formula:

(3) $$\frac{OD\;positive\;control-OD\;sample}{OD\;positive\;control}\times 100\%$$

#### 2.9.2. Live and Dead Bacteria Assay

To assess the viability of MRSA exposed to various concentrations of SA-AgNPs, live/dead bacterial assays were performed using confocal laser scanning microscopy (FV3000, Olympus, Tokyo, Japan). MRSA was cultured overnight in an LB medium. The bacterial suspensions were washed in sterile PBS (pH = 7.4) three times to remove the medium and then resuspended in sterile PBS to OD_600_ of 2.0. The bacteria were incubated with SA-AgNPs (1:1, *v*/*v*) at 37 °C for 1 h. Following this step, the suspension was stained and incubated at room temperature for 15 min, shielded from light. Bacterial viability was observed using the SYTO 9/propidium iodide (PI) double-stain kit (Maokang, Shanghai, China), following the guidelines provided by the manufacturer.

### 2.10. Determination of Leakage of DNA, RNA, and Protein Concentrations in Bacterial Cell

The bacteria (MRSA) were washed and resuspended in an M9 minimal medium (containing 10 mM NaAc, 2 mM MgSO_4_, 0.1 mM CaCl_2_). OD_600_ of the bacterial suspension was set to 0.6, then incubated with different concentrations (8 × MIC and 16 × MIC) SA-AgNPs at 37 °C and 160 rpm for 6 h, respectively. Bacteria without SA-AgNPs treatment as a control group. The supernatant was isolated by centrifugation (8000× *g*) for 5 min at 4 °C. The concentrations of DNA and RNA leaked from MRSA were measured at 260 nm by NanoDrop 2000 UV–vis spectrophotometer (Thermo Fisher Scientific, Waltham, MA, USA). The concentrations of proteins in the supernatant were determined using the dilution-free rapid gold BCA protein assay kit (Thermo Fisher Scientific, Waltham, MA, USA). The absorbance was measured at 490 nm.

### 2.11. Statistical Analysis

The study was conducted using three independent experimental groups. All findings reported in this research are presented as mean values accompanied by their standard deviations (SD). Data analysis was performed using Origin 2021 (OriginLab, Northampton, MA, USA) and GraphPad Prism 8.0.1 (GraphPad Software Inc., San Diego, CA, USA). Statistical comparisons were made using both one-way and two-way analysis of variance (ANOVA). *p* < 0.05 indicated the difference was statistically significant. * represents *p* < 0.05; ** represents *p* < 0.01; *** represents *p* < 0.001; **** represents *p* < 0.0001.

## 3. Results and Discussion

### 3.1. Green Synthesis of Salicylic Acid-Silver Nanoparticles (SA-AgNPs)

SA and AgNO_3_ were sonicated at room temperature, resulting in a dark brown solution. After changing the pH of the AgNO_3_ solution and adding salicylic acid for ultrasonication, the sample solution changed from colorless and translucent to dark brown (Figure S1A,B), indicating that the silver ions were reduced to Ag^0^ and silver nanoparticles were generated [24,43]. The sample was subsequently centrifuged, rinsed three times with ultrapure water, and freeze-dried to yield a brown-black powder (Figure S1C).

In this study, salicylic acid was used as a reducing and capping agent. AgNO_3_ was utilized as a precursor for the AgNPs, ultrapure water as a solvent for dissolution, and sodium hydroxide to modify pH. The entire synthesis process was easy and ecologically friendly. Phenolic chemicals can act as electron or hydrogen donors in the chemical reaction that produces AgNPs [31,44]. Unlike previous studies relying on complex plant extracts [45,46,47,48,49], our method employs pure SA to ensure reproducibility and mechanistic clarity.

### 3.2. Study of the Effect of Different Factors on the Biosynthesis of SA-AgNPs

#### 3.2.1. Effect of Ultrasonic Time

**Figure 1:**
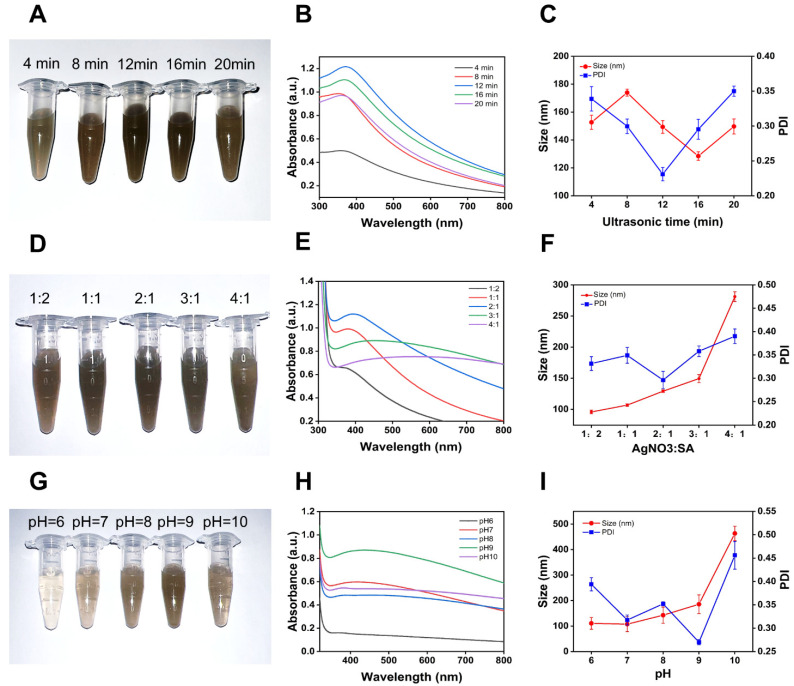
(**A**) Photograph of SA-AgNPs synthesized at different ultrasonic times, (**D**) of SA-AgNPs synthesized at different volume ratios, and (**G**) of SA-AgNPs synthesized at various pH. (**B**) UV-vis spectra of SA-AgNPs produced at various ultrasonic times, (**E**) of SA-AgNPs synthesized at various volume ratios, and (**H**) of SA-AgNPs synthesized at different pH. (**C**) Particle size and PDI of SA-AgNPs synthesized at various ultrasonic times, (**F**) of SA-AgNPs synthesized at different volume ratios, and (**I**) of SA-AgNPs synthesized at different pH.

As demonstrated in Figure 1A, SA-AgNPs were synthesized at various ultrasonic times (4, 8, 12, 16 and 20 min). By comparing the UV-Vis spectra corresponding to the SA-AgNPs solutions, Figure 1B makes it clear that the peak intensity of the SPR absorption bands reaches its maximum when the ultrasonic time is 12 min. After the ultrasonic time exceeds 12 min, the peak intensity decreases with further increases in ultrasonic duration, while the wavelength of the SPR peaks remains relatively unchanged. This suggests that ultrasonic treatment has little effect on the size of SA-AgNPs, aligning with hydrodynamic size measurements [50].

The SA-AgNPs particle size ranged from 120 nm to 180 nm during the 4–20 min ultrasonication interval, as shown in Figure 1C. After 12 min of sonication, the SA-AgNPs particle size was 149.37 ± 4.55 nm, and their PDI was the least, measuring less than 0.3 (0.231 ± 0.010). Thus, one of the ideal experimental settings is a 12-min ultrasonic time.

#### 3.2.2. Effect of Volume Ratio

SA-AgNPs were synthesized with various volume ratios (1:2, 1:1, 2:1, 3:1, and 4:1), as shown in Figure 1D. The volume ratio (AgNO_3_:SA) was the primary experimental element affecting the concentration and size of SA-AgNPs. This was verified by the UV-vis spectra of SA-AgNPs synthesized with different volume ratios. Figure 1E demonstrates that the peak intensity of the SPR peak reaches its maximum when the volume ratio (AgNO_3_:SA) is increased from 1:2 to 2:1. The peak intensity of the SPR peak diminishes as the volume ratio (AgNO_3_:SA) increases from 2:1 to 5:1. This implies that the maximum concentration of nanoparticles was generated using a volume ratio of 2:1 (AgNO_3_:SA).

Furthermore, when the volume ratio (AgNO_3_:SA) was higher than 2:1, the SPR peak wavelength redshifted, and the peak shape broadened and flattened, indicating that the particle size of SA-AgNPs increased [51]. In Figure 1F, SA-AgNPs had a particle size of 129.2 ± 1.98 nm at a 2:1 volume ratio (AgNO_3_:SA) and a PDI of 0.296 ± 0.018. The optimal volume ratio is (AgNO_3_:SA) 2:1.

#### 3.2.3. Effect of pH

SA-AgNPs were generated at various pH levels (6, 7, 8, 9, and 10), as indicated in Figure 1G. pH significantly influences silver nanoparticles’ formation and dimensions [49,52]. Figure 1H demonstrates that SPR peaks did not form in the UV-Vis spectra when the solution was acidic (pH = 6). The SPR peaks began to appear at pH 7 and reached their maximum intensity as the pH climbed to 9. Combined with Figure 1I, at pH 10, the peak intensity decreased again while the particle size and PDI increased, demonstrating that too high a pH not only decreases the creation of SA-AgNPs but also increases their size and induces the nanoparticles to assemble [49].

The solution’s absorption intensity was highest at pH 9, with an even dispersion of nanoparticles (PDI = 0.270 ± 0.006). As a result, the optimal pH was 9. Similar results have been obtained in previous studies [53,54,55,56]. It also has demonstrated that the optimal pH for green synthesis of silver nanoparticles varies with the reducing agent [57].

### 3.3. Characterization of SA-AgNPs

#### 3.3.1. UV-Visible Spectrophotometer Analysis

**Figure 2:**
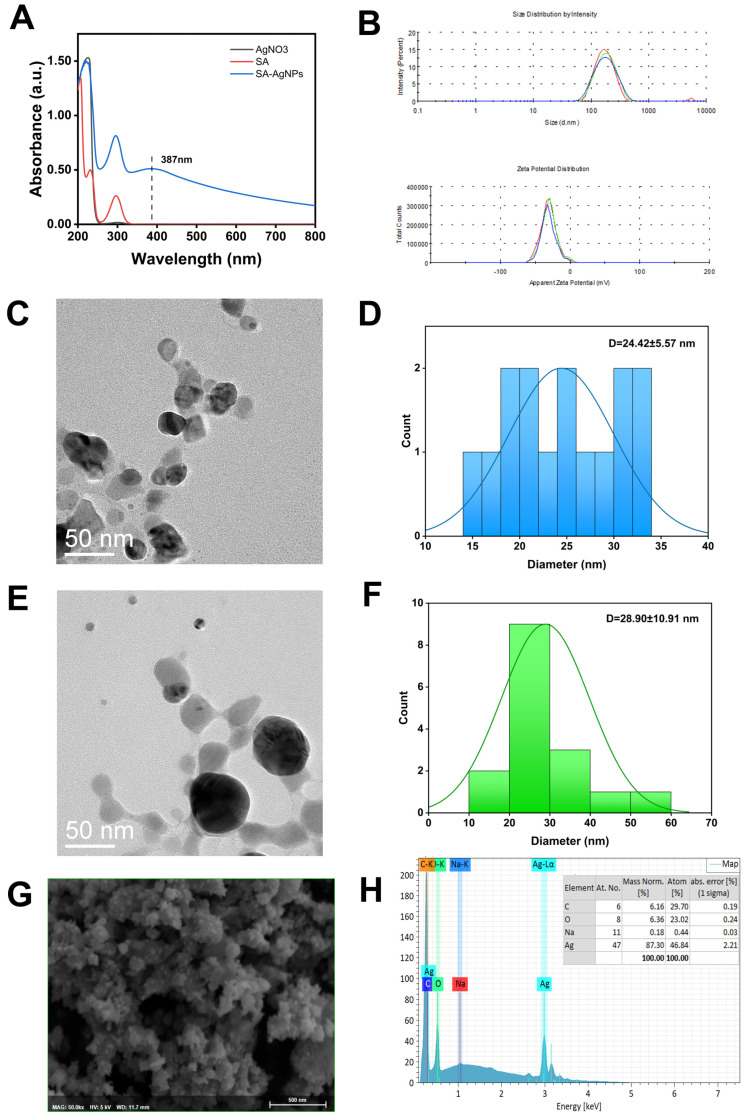
(**A**) UV-Vis spectrum for SA-AgNPs, SA, and AgNO3. (**B**) particle size and zeta potential of SA-AgNPs. (**C**,**E**) TEM images of SA-AgNPs. (**D**,**F**) diameter measurements of SA-AgNPs represented as histogram graphs. (**G**,**H**) SEM-EDX images of SA-AgNPs.

UV-Vis Spectrophotometer is an important tool for characterizing the optical properties of AgNPs. Because of their unique optical properties, metal nanoparticles form SPR peaks that can be absorbed between 350 and 500 nm. As shown in Figure 2A, SA and AgNO_3_ have no absorbance peaks near 400 nm, while the synthesized SA-AgNPs exhibit a new absorbance peak at 387 nm. This is a typical SPR band for silver nanoparticles, indicating their formation [58].

Based on Mie-type scattering theory, the UV-Vis peak position reveals the size of AgNPs [50]. The narrower the shape of AgNPs’ SPR peak, the more evenly distributed they are, and vice versa [59]. A blueshift of SPR bands shows a decrease in the particle size of the AgNPs generated, while a redshift of SPR bands indicates an increase in particle size [51]. A higher SPR peak absorption value indicates a higher concentration of AgNPs in the solution [60]. This principle was applied in single-factor experiments and confirmed by hydrodynamic particle size and PDI measurements.

#### 3.3.2. Hydrodynamic Particle Size and Zeta Potential Measurement

The diluted SA-AgNPs samples’ hydrodynamic particle size and zeta potential were determined by dynamic light scattering (DLS), and the results are given in Figure 2B. The SA-AgNPs produced under optimal experimental conditions had a hydrodynamic particle size of 166.8 nm, a zeta potential of −31 mV, and a PDI of 0.237, as determined by a Zetasizer Nano ZS instrument. The PDI of the SA-AgNPs was less than 0.3, indicating that the silver nanoparticles were distributed equally. The higher the absolute value of the zeta potential, the stronger the repulsion between particles and the lower the chance of aggregation [61]. As a result, SA-AgNPs are difficult to aggregate in solution and remain rather stable. Additionally, for stronger antibacterial action, SA-AgNPs’ negative charge on the surface helps them adhere to germs.

#### 3.3.3. Transmission Electron Microscopy (TEM)

The size and surface morphology of SA-AgNPs were observed using TEM. According to Figure 2C,E, SA-AgNPs have a nearly spherical form with some particle agglomeration, which could be brought on by freeze-drying. Particle size statistics of SA-AgNPs in TEM images were analyzed using ImageJ 2.0. Figure 2D,F show histograms of the average diameter of the SA-AgNPs, with average particle sizes of 24.42 ± 5.57 nm and 28.90 ± 10.91 nm.

The particle sizes determined by the DLS and the ones counted in the TEM pictures differ significantly. This is because the SA-AgNPs’ hydrodynamic particle size in a liquid (water) is measured by DLS. The hydrodynamic particle size usually appears much larger than the TEM particle size [62], which is the actual particle size. Since the hydrodynamic size comprises not only the inorganic particles in the core but also the hydration layer on the surface and the attached organic components, the hydration layer on the AgNPs’ surface is what causes the difference between the two.

#### 3.3.4. Scanning Electron Microscope-Energy Dispersive X-Ray Spectroscopy (SEM-EDX)

The surface morphology and elemental composition of SA-AgNPs were determined using SEM-EDX. Based on Figure 2G,H and Figure S2, the SA-AgNPs are elliptical or sub-spherical in shape and primarily composed of Ag. EDX spectra (Figure S3) revealed the presence of C and O, indicating that SA successfully capped AgNPs. Silver nanoparticles typically have a strong signal peak at 3 keV because of SPR. This shows the existence of silver as a component element, verifying the synthesis of SA-AgNPs. SA-AgNPs contain the largest atomic percentage of Ag (46.84%), followed by C (29.70%) and O (23.02%). C and O are obtained from salicylic acid, while O may also be derived from airborne contaminants adsorbed on the samples’ surfaces. The presence of C and O atoms verifies salicylic acid’s role as a capping and stabilizing agent on the surface of silver nanoparticles [63]. The remaining very minor amount of sodium (0.44%) came from sodium hydroxide.

#### 3.3.5. X-Ray Diffraction Analysis

**Figure 3:**
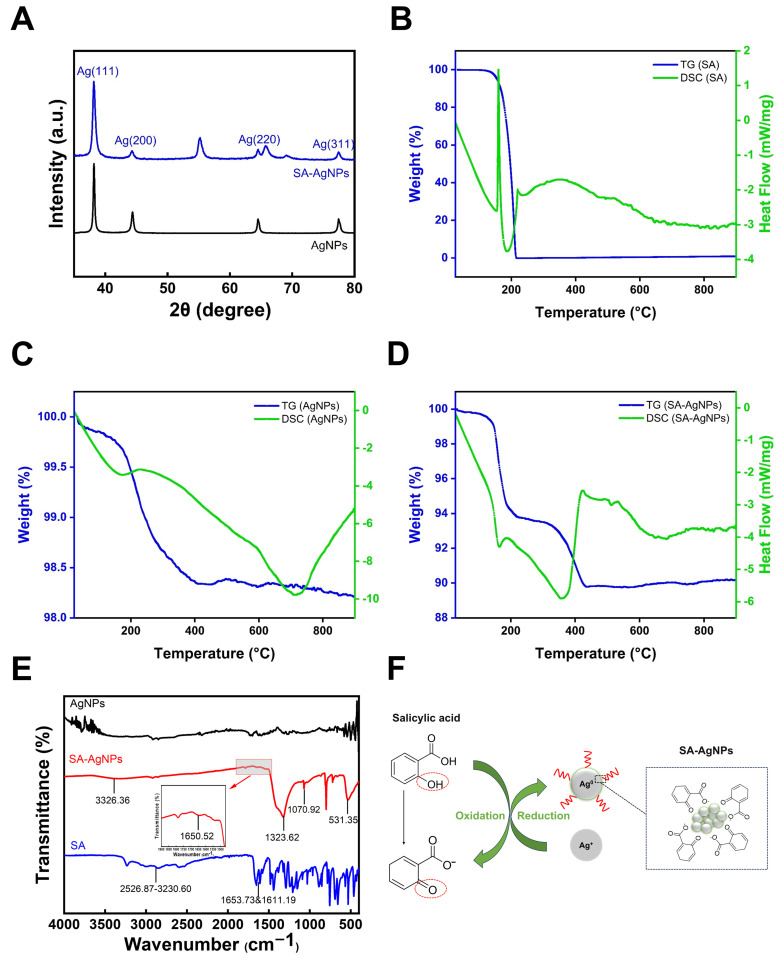
(**A**) XRD spectra of SA-AgNPs. (**B**) TG-DSC results for SA, (**C**) for commercial AgNPs, and (**D**) for SA-AgNPs. (**E**) FTIR spectrum (SA-AgNPs, SA and commercial AgNPs). (**F**) Possible mechanism of SA-AgNPs formation.

Figure 3A shows the XRD results of synthesized SA-AgNPs. SA-AgNPs exhibited four distinct diffraction peaks at 38.12°, 44.22°, 64.50°, and 77.35°, which correspond to lattice planes (111), (200), (220), and (311), respectively. These peaks are also consistent with the four distinct peaks of commercial AgNPs at 38.12°, 44.35°, 64.46°, and 77.39°. The XRD spectrum of the SA-AgNPs was compared to the Joint Committee on Powder Diffraction Standards (JCPDS) file (No. 04–0783), which revealed the crystalline nature of SA-AgNPs [64].

However, there is a little shift in peak positions, which could be due to strain in the crystal structure. The peaks measured at 55.19° and 69.04° are similar to those of silver oxide [63]. The sharp peaks indicate strong crystallinity in AgNPs. This result validates the presence of silver in the prepared samples, as well as the crystallinity of SA-AgNPs.

#### 3.3.6. Thermogravimetric-Differential Scanning Calorimetry (TG-DSC)

The thermal stability of SA-AgNPs was evaluated with TG-DSC. TG-DSC results are displayed in Figure 3B–D. As seen in the picture, the SA powder loses mass by approximately 100% after 215 °C, which is related to the fact that SA is an organic compound. The weight loss of commercial AgNPs without SA capping was approximately 1.70% between 32 °C and 407 °C. SA-AgNPs lost approximately 6.23% between 32 °C and 221 °C, and 3.98% between 221 °C and 434 °C, possibly due to water evaporation and loss of organic matter on the silver nanoparticles’ surfaces [65].

The overall weight loss of SA-AgNPs was 10.21%, and the melting point of silver is 960.54 °C [66], implying that the weight loss in the tested range is not due to silver; hence, the silver content can be determined to be 89.79%. This is consistent with the silver content estimated by EDX (87.30 ± 2.21%).

#### 3.3.7. Fourier Transform Infrared Spectroscopy (FTIR)

IR analysis was applied to identify potential functional groups and formation mechanisms in SA-AgNPs. Figure 3E presents a comparison between the blank samples (commercial AgNPs and salicylic acid) and SA-AgNPs.

As demonstrated, the broad O–H stretching band of salicylic acid at 2526.87–3230.60 cm^−1^ results from the superposition of carboxylic acid and phenolic hydroxyl groups [67]. The two strong peaks at 1653.73 cm^−1^ and 1611.19 cm^−1^ represent the carbonyl group in salicylic acid.

The O–H stretching band of SA-AgNPs shifted to 3326.36 cm^−1^ compared to salicylic acid, indicating the loss of proton hydrogen [67]. This suggests that the O–H stretching of SA was likely to contribute to the synthesis of AgNPs [63]. The peak at 1650.52 cm^−1^ is attributed to the stretching of C=O groups or C=C stretching of aromatic ring [68]; the peak at 1323.62 cm^−1^ may be due to C–O stretching vibration in phenols [69]; the peak at 1070.92 cm^−1^ corresponds to the C–C or C–O stretching [70]; and the band at 531.35 cm^−1^ can be ascribed to the transition-metal Ag energy band [71]. The IR spectra suggest that the phenolic hydroxyl group (–OH) in salicylic acid reduces Ag^+^ to Ag^0^ while the phenolic hydroxyl group on the benzene ring is oxidized [22,72]. The capping alteration of AgNPs by salicylic acid was eventually achieved. According to the literature, phenolic compounds can serve as reducing and capping agents, enabling the synthesis of stable silver nanoparticles [73]. The phenolic hydroxyl groups in phenolic acids ionize under alkaline conditions to form phenolate ions, which act as electron donors to reduce Ag⁺ ions [74,75]. This redox process simultaneously oxidizes the phenolate into a quinonoid form [74,76]. Figure 3F illustrates a possible mechanism of SA-AgNPs formation according to the IR spectra.

### 3.4. Assay of Salicylic Acid

The content of SA was determined using the RP-HPLC technique. Linearity was seen at concentrations between 0.01 and 0.15 mg/mL. The sample solution’s peak area was estimated using a linear equation (Figure S4), yielding the SA content in the supernatant of 68.09%. Specifically, the total mass of SA is 1.0 mg, so the precipitated SA-AgNPs contained 0.3191 mg of SA, indicating that 31.91% of total SA was attached to the silver nanoparticles’ surface.

### 3.5. Assay of Silver Ions

SA-AgNPs had 90.61 ± 1.57% (Wt%) silver, as determined by ICP-AES analysis. This result corresponds to the EDX measurement of 87.30 ± 2.21% silver and the TG-DSC result of 89.79% silver.

### 3.6. Stability of SA-AgNPs

**Figure 4:**
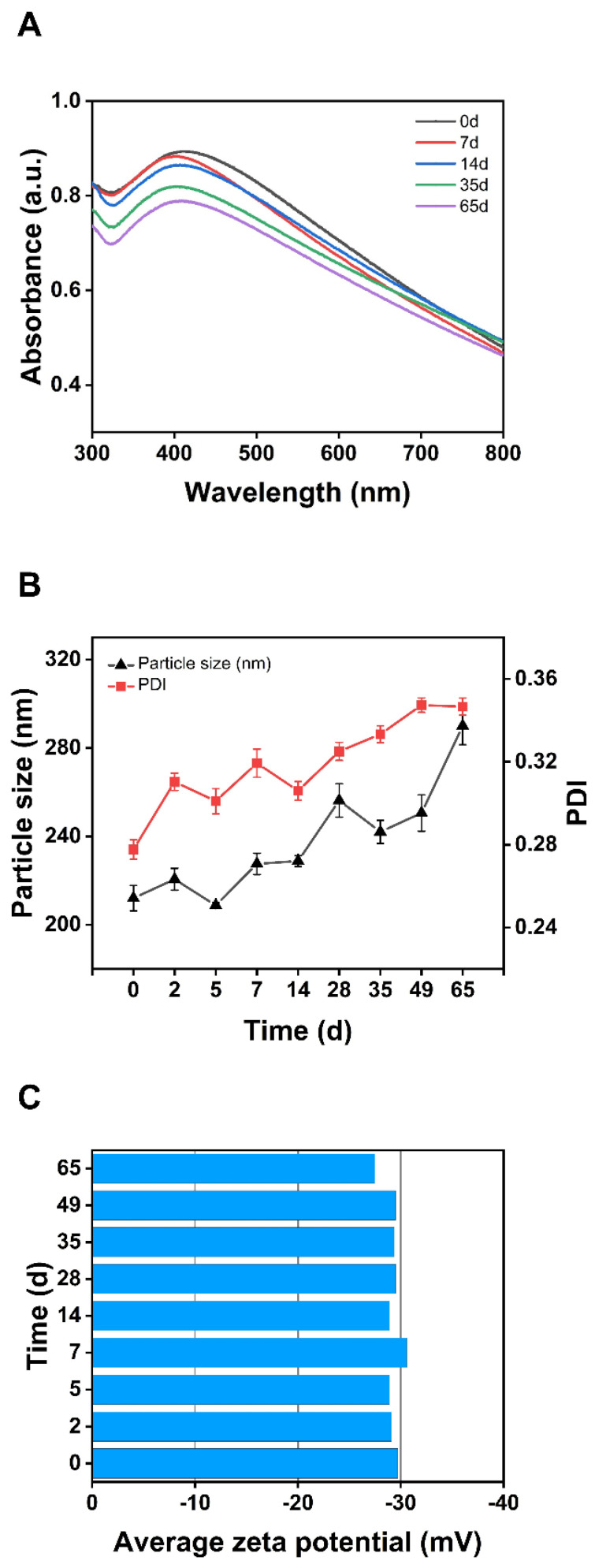
(**A**) UV-vis spectra of SA-AgNPs on different days. (**B**) Particle size and PDI of SA-AgNPs on different days. (**C**) Zeta potential of SA-AgNPs at different days.

Stability tests are an essential step before the further application of nanoparticles. Thus, the stability of SA-AgNPs at 4 °C was investigated. UV-vis spectrophotometry can identify changes in the SPR peaks of silver nanoparticles, while PDI and zeta potential analyses reflect their stability in solution [38]. The higher the absolute value of the potential and the lower the PDI value, the more stable SA-AgNPs are in the solution [77,78]. As indicated in Table 1 and Figure 4A, the wavelength and peak intensity of SA-AgNPs’ SPR peak changed very little over 65 days. Figure 4B demonstrates an overall increase in particle size and PDI of SA-AgNPs, suggesting that nanoparticles may have agglomerated over this period. However, Figure 4C demonstrates that the zeta potential of SA-AgNPs remains almost unaltered after 65 days, and the absolute value is high. As a result, SA-AgNPs maintain good stability at 4 °C for 65 days.

### 3.7. Antibacterial Activity of SA-AgNPs

#### 3.7.1. Minimum Inhibitory Concentration (MIC) and Minimum Bactericidal Concentration (MBC)

Measuring MIC and MBC determined the minimal concentrations at which SA-AgNPs inhibited bacterial growth and killed bacteria. The MIC and MBC for SA-AgNPs, SA, commercial AgNPs, and ampicillin against various bacteria are shown in Table 2 and Table 3, respectively.

The MIC and MBC of SA-AgNPs for MRSA and *S. aureus* are recorded at 8 μg/mL and 16 μg/mL, respectively. For *E. coli*, the MIC and MBC values stand at 6 μg/mL and 12 μg/mL. When it comes to MRSE, the MIC is 4 μg/mL while the MBC is 8 μg/mL. For *C. acnes*., the MIC and MBC are 9 μg/mL and 18 μg/mL, respectively. The MICs of SA-AgNPs against *S. aureus* and MRSA were approximately half that of AgNPs synthesized by *Scutellaria baicalensis* polysaccharide [79], indicating that SA-AgNPs have excellent antibacterial properties.

Salicylic acid (≥800 μg/mL) and commercial AgNPs (≥64 μg/mL) had significantly higher MIC values than synthetic SA-AgNPs. For the drug-resistant bacteria MRSE, the MIC of SA-AgNPs is lower than that of the positive drug ampicillin.

According to the MIC and MBC values, SA-AgNPs had significantly better bacteriostatic and antibacterial activity than those of salicylic acid and commercial AgNPs. This indicates the synergistic action of AgNPs combined with salicylic acid. The study found that the size, shape, concentration, and exposure period of silver nanoparticles influence their bactericidal action [80]. Furthermore, silver nanoparticles are more efficient at killing Gram-negative bacteria than Gram-positive bacteria, primarily because Gram-positive bacteria have thicker cell walls [81]. The antibacterial property of silver nanoparticles is primarily achieved by acting on the cell membrane and increasing its permeability; thus, thick cell walls reduce its antimicrobial activity.

#### 3.7.2. Time-Kill Assay

**Figure 5:**
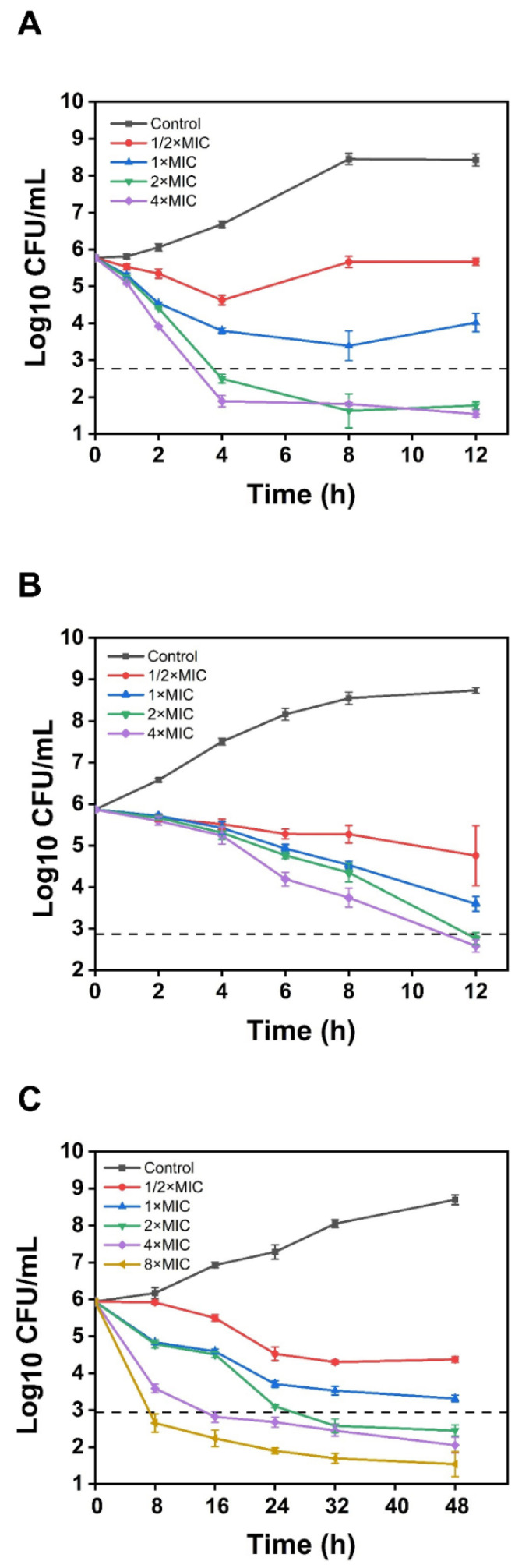
(**A**) Time-kill curves for various concentrations of SA-AgNPs against *E. coli*. (**B**) Time-kill curves for various concentrations of SA-AgNPs against MRSA. (**C**) Time-kill curves for various concentrations of SA-AgNPs against *C. acnes*.

Time-kill assays were conducted to investigate the killing effect of Gram-positive bacteria (MRSA and *C. acnes*) and Gram-negative bacteria (*E. coli*) at various concentrations of SA-AgNPs (1/2 × MIC, MIC, 2 × MIC, and 4 × MIC). Figure 5A–C exhibits the bacterial concentration (log_10_ CFU/mL) versus time. SA-AgNPs at 2 × MIC and 4 × MIC levels killed *E. coli* and MRSA within 4 and 12 h, respectively, which means the bactericidal rate reached 99.9%. SA-AgNPs at 2 × MIC, 4 × MIC, and 8 × MIC concentrations killed *C. acnes* within 32 h, 16 h, and 8 h, respectively. SA-AgNPs (2 × MIC and 4 × MIC) killed *E. coli* in just 4 h, faster than killing Gram-positive bacteria (MRSA and *C. acnes*). Compared to other green synthesized silver nanoparticles, SA-AgNPS showed significantly higher bactericidal efficiency against MRSA. After 24 h of treatment with silver nanoparticles-kaempferol (1.25 mg/mL), MRSA growth decreased by 1.30 log_10_CFU/mL [24], while SA-AGNPS (16 µg/mL) reduced growth by 3.1 log_10_CFU/mL in 12 h.

### 3.8. Antibiofilm Ability Studies

#### 3.8.1. Biofilm Formation Inhibition

**Figure 6:**
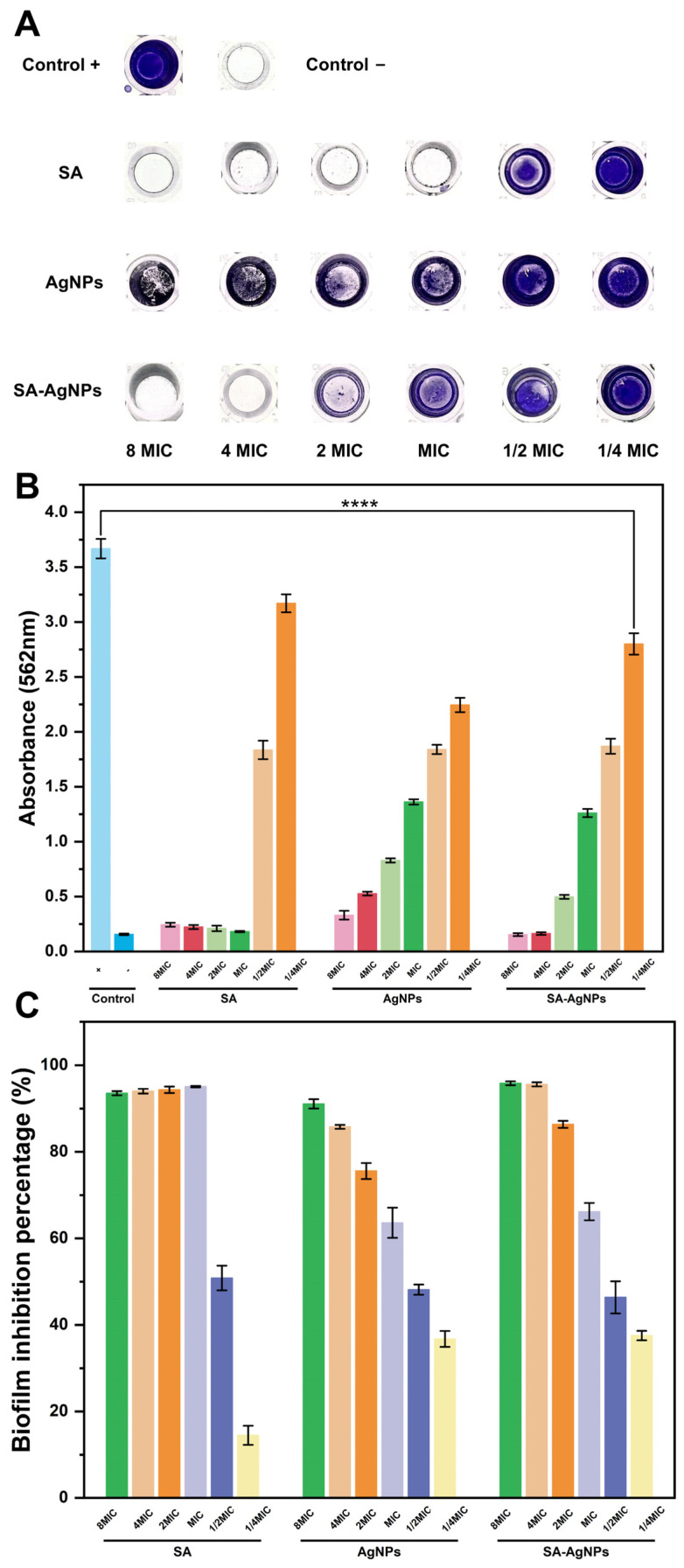
(**A**) Images of residual biofilm of MRSA. (**B**) Effects of different SA, AgNPs, and SA-AgNPs concentrations on MRSA biofilm formation. **** represents *p* < 0.0001. (**C**) Biofilm inhibition percentage of MRSA treated with various concentrations of SA, AgNPs, and SA-AgNPs.

Biofilms serve as a critical factor in the development of more than 80% of bacterial infections [82], playing a crucial role in the defense against antibacterial agents. Therefore, inhibiting biofilm formation effectively can reduce drug-resistant bacterial infections. Crystal violet dye (0.1%) was used to quantitatively determine the biofilm formation of MRSA treated with various concentrations of SA, AgNPs, and SA-AgNPs (Figure 6A–C). As shown in Figure 6B, SA-AgNPs at the lowest concentration of 1/4 × MIC (2 μg/mL) significantly reduced MRSA biofilm formation (*p* < 0.0001). Additionally, SA, AgNPs, and SA-AgNPs inhibited biofilm formation in MRSA in a concentration-dependent manner. The negative control wells had a mean OD of 0.1558 and a standard deviation of 0.0072, resulting in an ODc of 0.1774. Biofilm production was categorized as follows: OD ≤ ODc, no biofilm; ODc < OD ≤ 2 × ODc, weak biofilm; 2 × ODc < OD ≤ 4 × ODc, moderate biofilm; and OD > 4 × ODc, strong biofilm [83]. According to Figure 6B, SA at 6.4, 3.2, 1.6, and 0.8 mg/mL caused weak biofilm, while 0.4 and 0.2 mg/mL caused strong biofilm. AgNPs at 2048 and 1024 μg/mL caused weak and moderate biofilm, respectively, and at 512, 256, 128, and 64 μg/mL caused strong biofilm. SA-AgNPs at 64 and 32 μg/mL resulted in no biofilm, 16 μg/mL caused moderate biofilm, and 8, 4, and 2 μg/mL caused strong biofilm.

As shown in Figure 6C, SA at the concentrations of 6.4, 3.2, 1.6, 0.8, 0.4, and 0.2 mg/mL decreased biofilm formation by 93.56%, 94.03%, 94.35%, 95.09%, 50.84%, and 14.50%, respectively. AgNPs at 2048, 1024, 512, 256, 128, and 64 μg/mL decreased biofilm formation by 91.10%, 85.79%, 75.59%, 63.63%, 48.16%, and 36.77%, respectively. SA-AgNPs at 64, 32, 16, 8, 4, and 2 μg/mL reduced biofilm formation by 95.86%, 95.61%, 86.35%, 66.17%, 46.37%, and 37.55%, respectively. Compared to SA and AgNPs, SA-AgNPs had superior anti-biofilm activity at lower concentrations (64 and 32 μg/mL), completely inhibiting biofilm formation. At about the same dose, AgNPs using the extract of aerial parts of the Anthemis pseudocotula Boiss. plant (39 μg/mL) inhibits biofilm formation of MRSA by only 67.81% [84], whereas SA-AgNPs (32 μg/mL) inhibit 95.61%.

#### 3.8.2. Live and Dead Bacteria Assay

**Figure 7:**
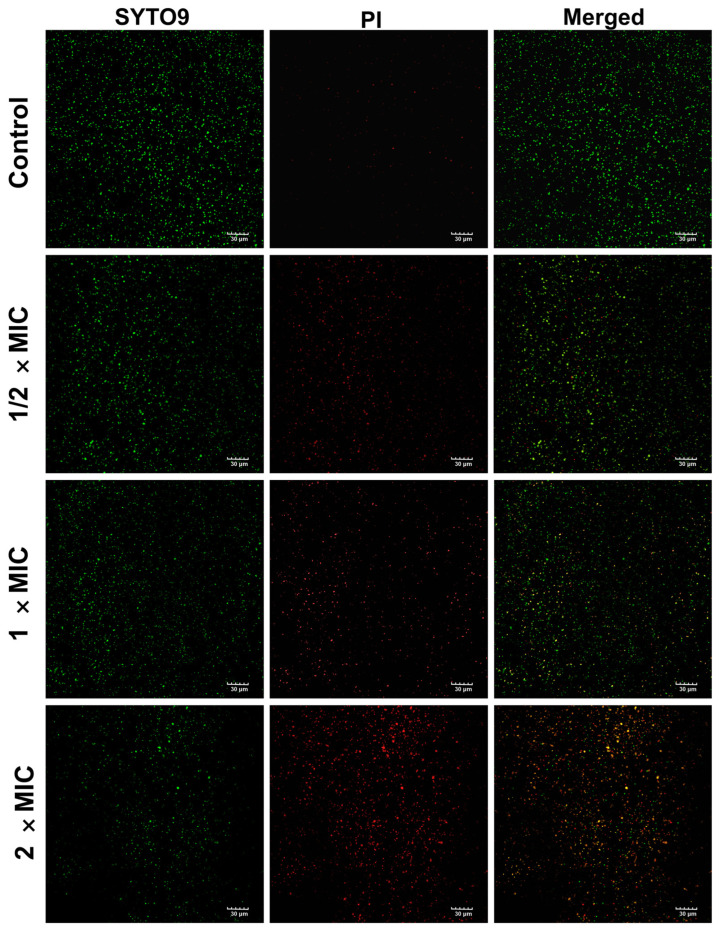
The confocal laser scanning microscopy images of MRSA were treated with SA-AgNPs (1/2 × MIC, 1 × MIC and 2 × MIC) and sterile PBS (control), respectively.

SYTO 9 penetrates both intact and damaged bacterial cell membranes, attaching to DNA and emitting green fluorescence, whereas PI can only enter cells with broken membranes and emit red fluorescence. As demonstrated in Figure 7, untreated MRSA showed extensive green fluorescence and limited red fluorescence, indicating a high density of live cells with intact membranes [79]. Compared to the control group, SA-AgNPs treatment resulted in a decrease in green fluorescence, with wide areas of red and orange fluorescence (red-green fluorescence overlap), indicating a large number of apoptotic cells. In addition, as the concentration of SA-AgNPs grew, green fluorescence reduced, and red fluorescence increased. In conclusion, SA-AgNPs significantly increased bacterial death, preventing biofilm formation.

### 3.9. Effect of SA-AgNPs on the Leakage of DNA, RNA, and Protein in Bacterial Cell

**Figure 8:**
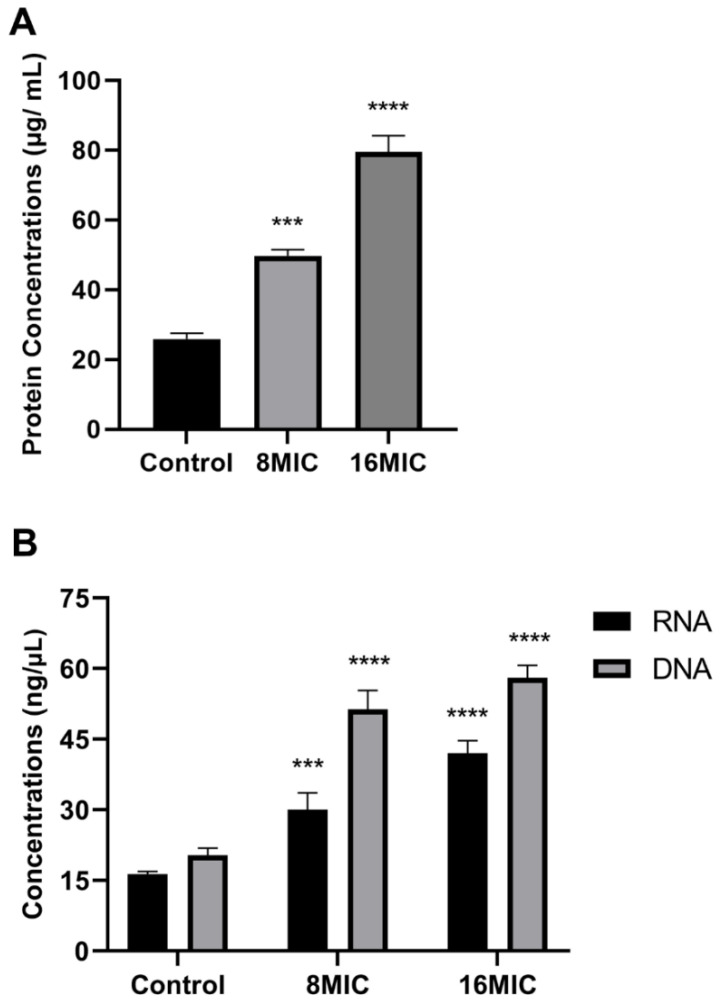
(**A**) Protein leakage in MRSA after 6 h of treatment with SA-AgNPs. (**B**) DNA and RNA leakage in MRSA after 6 h of treatment with SA-AgNPs. *** represents *p* < 0.001, **** represents *p* < 0.0001.

The leakage of intracellular compounds reflects cell membrane permeability. Extracellular DNA, RNA, and protein levels were measured to evaluate the effect of SA-AgNPs on bacterial membrane permeability. As shown in Figure 8A, SA-AgNPs significantly increased protein leakage compared to the control. Similar results were observed for DNA and RNA leakage in Figure 8B. SA-AgNPs dose-dependently increased membrane permeability and compound leakage, which is one of its mechanisms of antibacterial action.

## 4. Conclusions

A green synthesis method for silver nanoparticles (SA-AgNPs) mediated by salicylic acid was developed successfully. A major benefit of this approach is its simplicity, requiring only two reagents (silver nitrate and salicylic acid), and it can be performed at room temperature and pressure. Additionally, it does not involve toxic chemicals, making it an environmentally friendly approach. SA acts as both a reducing agent and a bioactive ligand during the synthesis of silver nanoparticles. This unique dual role not only facilitates the reduction of silver ions to form AgNPs but also endows the nanoparticles with enhanced antibacterial properties. This study demonstrates that, despite SA’s poor solubility, its role in AgNP synthesis is unaffected and can be optimized to enhance the antibacterial activity of the nanoparticles.

Consequently, SA-AgNPs demonstrated superior antimicrobial properties, with MIC values ranging from 4 to 9 μg/mL. The antibacterial activity of SA-AgNPs was significantly enhanced, 33-fold (for *E. coli* and *C. acnes*), 32-fold (for MRSA and *S. aureus*), and 16-fold (for MRSE) higher than that of commercial AgNPs, respectively. Moreover, SA-AgNPs inhibited nearly 96% of MRSA biofilm formation at a lower concentration (64 μg/mL), showing a 60% increase compared to commercial AgNPs at the same concentration. The effect of SA-AgNPs on bacterial membrane permeability led to protein, DNA, and RNA leakage, ultimately leading to cell rupture and apoptosis. Silver nanoparticles show antibacterial and antibiofilm activity against drug-resistant bacteria and are less likely to develop resistance, making them a potential alternative therapy for multidrug-resistant bacterial infections.

However, silver nanoparticles confront several limitations in therapeutic applications, such as potential toxicity and impacts on the environment. Additionally, current studies are constrained by a narrow pathogen testing scope and lack validation in biologically complex systems, hindering translational relevance. Further research should focus on refining the silver nanoparticle preparation method and minimizing its toxicity, as well as investigating its possible application in antimicrobial coatings for medical devices or wound dressing materials.

In conclusion, SA-AgNPs, as an emerging antimicrobial agent, have great potential in addressing the problem of bacterial drug resistance due to their wide-ranging and effective antimicrobial ability; however, further studies are required to confirm their safe and effective clinical application.

## Figures and Tables

**Table 1:** Wavelengths and absorption values of SPR peaks of SA-AgNPs at different days.

	0 d	2 d	5 d	7 d	14 d	28 d	35 d	49 d	65 d
λ_max_ (nm)	411	415	406	405	407	406	408	407	409
Absorbance	0.894	0.883	0.878	0.883	0.865	0.842	0.837	0.82	0.789

**Table 2:** MIC values of SA-AgNPs, SA, commercial AgNPs and positive drug (ampicillin) against *C. acnes*, *E. coli*, MRSA, MRSE, and *S. aureus*, respectively.

Compound	MIC (µg/mL)				
	*C. acnes*	*E. coli*	MRSA	MRSE	*S. aureus*
SA-AgNPs	9	6	8	4	8
Salicylic Acid	1800	800	800	800	800
Commercial AgNPs	300	200	256	64	256
Ampicillin	0.5	-	>512	128	1

**Table 3:** MBC values of SA-AgNPs against *C. acnes*, *E. coli*, MRSA, MRSE, and *S. aureus*, respectively.

Compound	MBC (µg/mL)				
	*C. acnes*	*E. coli*	MRSA	MRSE	*S. aureus*
SA-AgNPs	18	12	16	8	16

## Data Availability

Data is contained within the article.

## Supplementary Materials

The following supporting information can be downloaded at: https://www.mdpi.com/article/10.3390/pharmaceutics17040532/s1, Figure S1. (A) Pre-reaction mixture, (B) mixture after reaction, and (C) freeze-dried powder of SA-AgNPs. Figure S2. SEM images of SA-AgNPs at different magnifications. Figure S3. Energy spectrum scanning element (Ag and O) of SA-AgNPs. Figure S4. The standard curve of SA between 0.01 and 0.15 mg/mL.
