# Supplementary material for: Salicylic Acid-Mediated Silver Nanoparticle Green Synthesis: Characterization, Enhanced Antimicrobial, and Antibiofilm Efficacy
Source: Pharmaceutics. 2025 Apr 18;17(4):532. doi: 10.3390/pharmaceutics17040532 (PMC12030525; doi:10.3390/pharmaceutics17040532)
Supplement: Supplementary file 1 [file pharmaceutics-17-00532-s001.zip › pharmaceutics-3561283-supplementary.pdf]

Supporting information

Salicylic Acid-Mediated Silver Nanoparticle Green Synthesis: Characterization, Antimicrobial, and Antibiofilm Properties

Jingqing Zhang, Yuxu Chen, Yuanyu Xu, Zhimin Zhao, Xinjun Xu[*]

School of Pharmaceutical Sciences, Sun Yat-sen University, Guangzhou 510006, China

[*]Corresponding author, E-mail address: xxj2702@sina.com

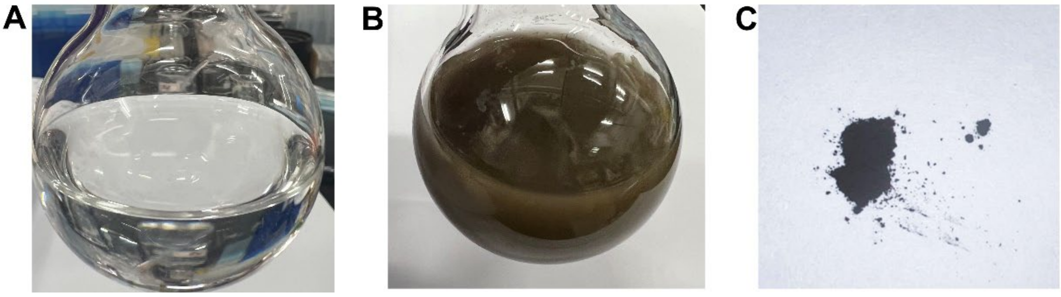

Figure S1. (A) Pre-reaction mixture, (B) mixture after reaction, and (C) freeze-dried powder of SA-AgNPs.

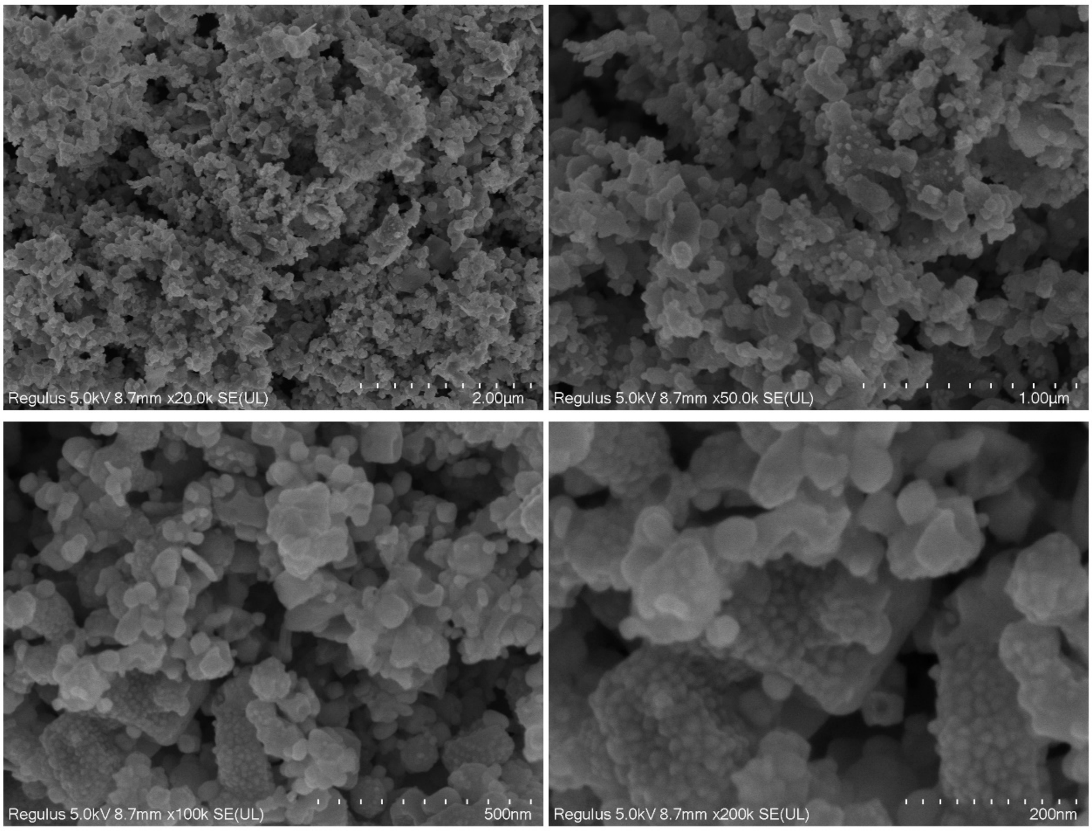

Figure S2. SEM images of SA-AgNPs at different magnifications.

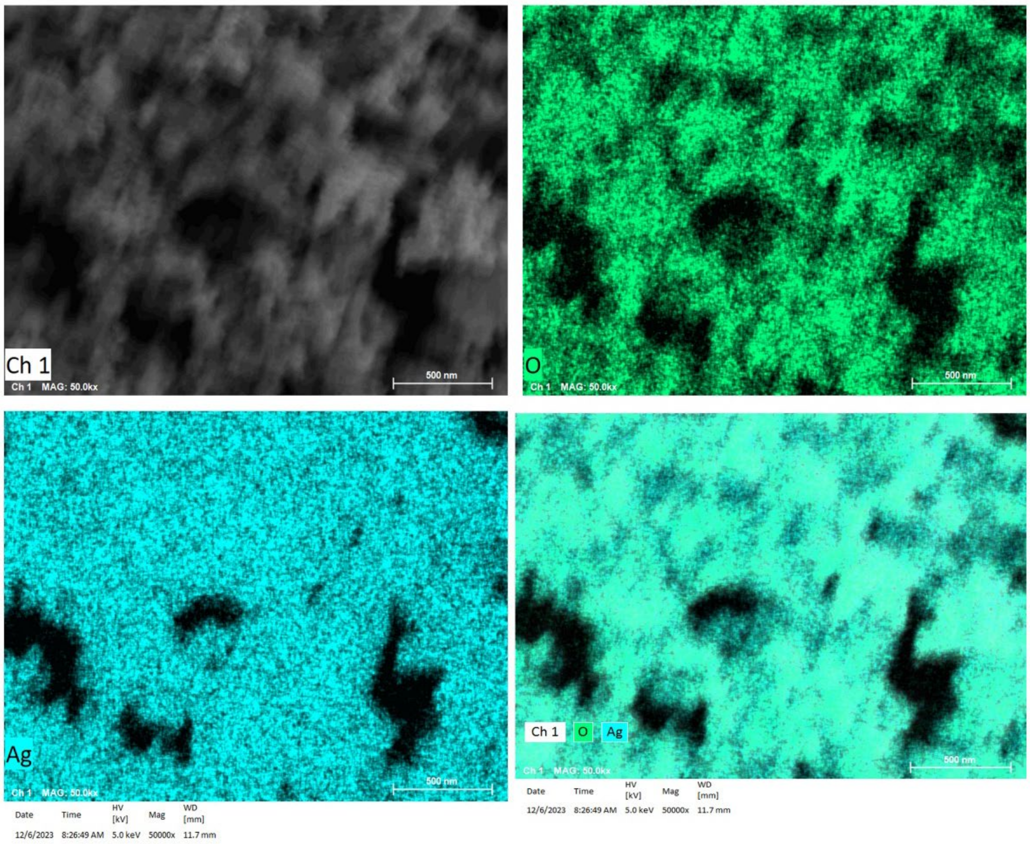

Figure S3. Energy spectrum scanning element (Ag and O） of SA-AgNPs

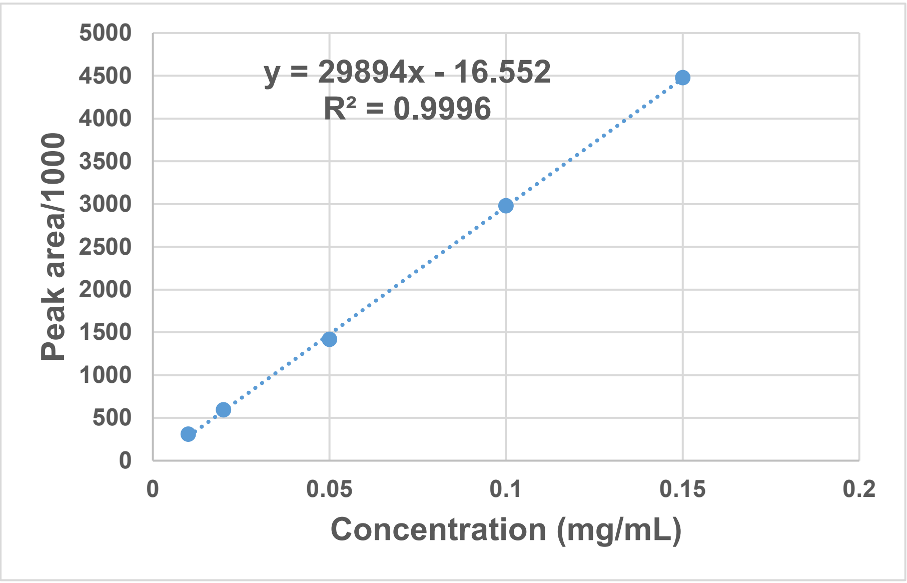

Figure S4. The standard curve of SA between 0.01 and 0.15 mg/ml.
